# Physicochemical Properties of a New PEGylated Polybenzofulvene Brush for Drug Encapsulation

**DOI:** 10.3390/pharmaceutics11090444

**Published:** 2019-09-01

**Authors:** Marco Paolino, Annalisa Reale, Vincenzo Razzano, Germano Giuliani, Alessandro Donati, Gianluca Giorgi, Antonella Caterina Boccia, Raniero Mendichi, Daniele Piovani, Chiara Botta, Laura Salvini, Filippo Samperi, Cristina Savoca, Mariano Licciardi, Eugenio Paccagnini, Mariangela Gentile, Andrea Cappelli

**Affiliations:** 1 Dipartimento di Biotecnologie, Chimica e Farmacia (Dipartimento di Eccellenza 2018–2022), Università degli Studi di Siena, Via Aldo Moro 2, 53100 Siena, Italy; 2 Istituto per lo Studio delle Macromolecole (CNR), Via A. Corti 12, 20133 Milano, Italy; 3 Toscana Life Sciences Foundation, Via Fiorentina 1, 53100 Siena, Italy; 4 Istituto per i Polimeri, Compositi e Biomateriali (IPCB) U.O.S. di Catania, CNR, Via Gaifami 18, 95126 Catania, Italy; 5 Dipartimento di Scienze e Tecnologie Biologiche, Chimiche e Farmaceutiche (STEBICEF), Università degli Studi di Palermo, Via Archirafi 32, 90123 Palermo, Italy; 6 Dipartimento di Scienze della Vita, Università degli Studi di Siena, Via Aldo Moro 2, 53100 Siena, Italy

**Keywords:** PEGylation, grafting through, polybenzofulvene, nanocarrier, drug delivery systems, spontaneous polymerization, affinity polymerization

## Abstract

A new polymer brush was synthesized by spontaneous polymerization of benzofulvene macromonomer 6-MOEG-9-T-**BF3k** bearing a nona(ethylene glycol) side chain linked to the 3-phenylindene scaffold by means of a triazole heterocycle. The polymer structure was studied by SEC-MALS, NMR spectroscopy, and MALDI-TOF MS techniques, and the results supported the role of oligomeric initiatory species in the spontaneous polymerization of polybenzofulvene derivatives. The aggregation features of high molecular weight poly-6-MOEG-9-T-**BF3k**-FE were investigated by pyrene fluorescence analysis, dynamic light scattering studies, and transmission electron microscopy, which suggested a tendency towards the formation of spherical objects showing dimensions in the range of 20–200 nm. Moreover, poly-6-MOEG-9-T-**BF3k**-FE showed an interesting cytocompatibility in the whole concentration range tested that, besides its aggregation features, makes this polybenzofulvene brush a good polymer candidate for nanoencapsulation and delivery of drug molecules. Finally, the photo-physical features of poly-6-MOEG-9-T-**BF3k**-FE could allow the biodistribution of the resulting drug delivery systems to be monitored by fluorescence microscopy techniques.

## 1. Introduction

Molecular brushes, polymer brushes, or cylindrical brushes are the most common names to designate a very intriguing large class of polymers obtained by dense grafting of polymeric side chains onto linear polymeric backbones [1,2]. The interesting features of this unique family of macromolecules are determined in a large part by side chain features such as length, physico-chemical properties, and grafting density. Furthermore, the conformational behavior of backbone and side chains can be affected by solvent properties, temperature, pH, and ionic strength so that polymer brushes (PB) can show macromolecular features sensitive to the surrounding environment [3]. They can be synthesized by means of three different procedures [3]: (a) grafting through (GT, macromonomer polymerizations) [4]; (b) grafting onto (GO, insertion of side chains onto polymeric backbones) [5]; and (c) grafting from (polymerization of side chains from a macroinitiator backbone) [6].

Around two decades ago, we discovered the spontaneous polymerization of benzofulvene derivative **BF1** (in the apparent absence of catalysts or initiators by evaporation of the solvent) and started a large program of synthetic and characterization studies, which led to the development of the large family of polybenzofulvene derivatives. These studies suggested for most of the synthesized polymers a vinyl structure stabilized by aromatic stacking interactions [7,8,9,10,11,12,13] and led to the obtainment of interesting materials showing remarkable optoelectronic features [14,15,16,17,18,19,20,21].

**Figure 1:**
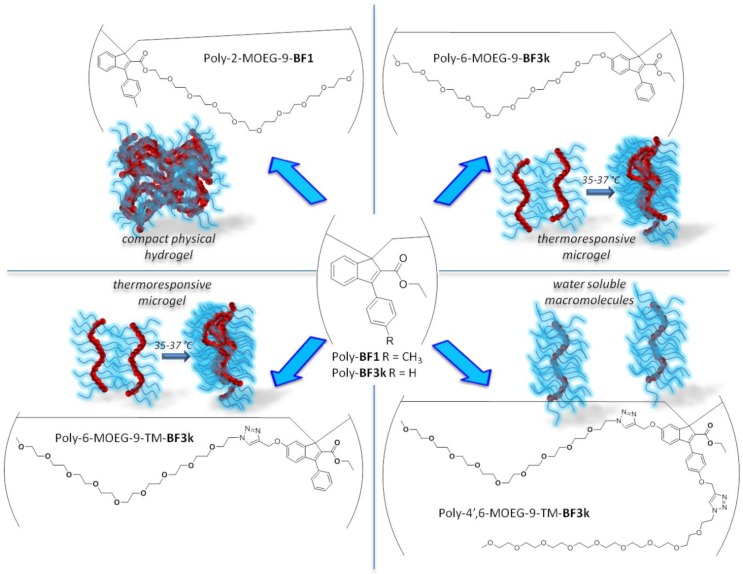
The previously proposed aggregation mechanism in PEGylated polybenzofulvene brush (PPBFB) derivatives.

By grafting monomethyl oligo(ethylene glycol) (MOEG) side chains (SC) onto the polybenzofulvene backbone of poly-**BF1** and poly-**BF3**, we designed and synthesized a family of PEGylated polybenzofulvene brushes (PPBFBs) that includes poly-2-MOEG-9-**BF1** [22], poly-6-MOEG-9-**BF3k** [23], poly-6-MOEG-9-TM-**BF3k** [24,25], poly-4,6-MOEG-9-TM-**BF3k** [26], and poly-4′,6-MOEG-9-TM-**BF3k** [26] (Figure 1) [27].

Intriguingly, these PPBFB derivatives were capable of interacting with water showing different aggregation features, which appeared to be modulated by the presence of the MOEG SC. In particular, the aggregation characteristics of the PPBFB derivatives spanned from the formation of a compact physical hydrogel (poly-2-MOEG-9-**BF1**) [28] to the water solubility of poly-4,6-MOEG-9-TM-**BF3k [26]** and poly-4′,6-MOEG-9-TM-**BF3k**, which showed reduced aggregation tendency due to the presence of two MOEG-9 side chains in each monomeric unit [26]. The results obtained supported the important role played by the presence and the location in the monomeric unit of MOEG-9 SC in the aggregation behavior of these PPBFB derivatives. 

Moreover, PPBFB derivatives were used in the development of drug delivery systems (DDS) such as the hydrogel obtained with poly-2-MOEG-9-**BF1** for the delivery of a human immunoglobulin [28] and the thermoresponsive gel nanoparticles obtained with poly-6-MOEG-9-**BF3k** for the delivery of the low molecular weight peptide anticancer drug leuprolide [29].

The biocompatibility and the apparent bioresorbability features of our PPBFB stimulated the employment of poly-6-MOEG-9-TM-**BF3k** architecture in the development of a PPBFB copolymer (i.e., poly-6-MOEG-9-TM-**BF3k**-co-6-BAUP-TM-**BF3k**) bearing dynamic binding sites [30,31,32] in order to obtain a macromolecule capable of loading the anticancer drug doxorubicin and releasing the drug to cancer cells [33]. The tri-component polybenzofulvene brush **TCPB** was then developed as a third-generation PPBFB, in which the core–shell architecture was further elaborated by the presence of the external targeting shell [34] consisting of low molecular weight hyaluronic acid (HA) macromolecules [34,35]. We assumed that the HA coating of **TCPB** was capable of interacting with CD44 receptors (overexpressed in many tumor cells) and therefore mediating the targeted delivery of doxorubicin to tumor cells [36]. 

**Figure 2:**
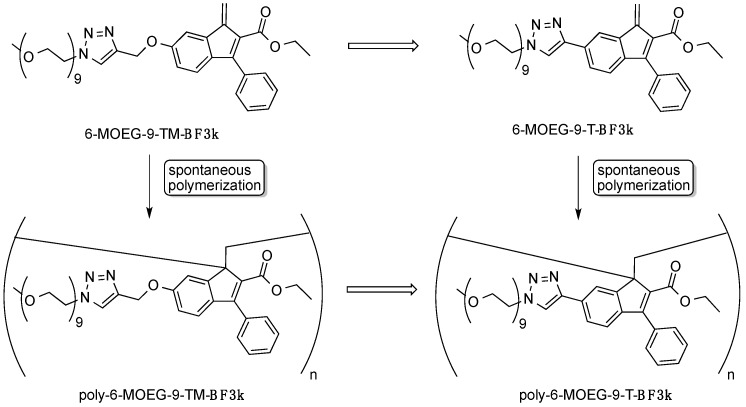
Design of PPBFB poly-6-MOEG-9-T-**BF3k** from poly-6-MOEG-9-TM-**BF3k**.

In the aim of evaluating the role of the methylene-oxy spacer in the aggregation features of these PEGylated polybenzofulvene brushes, the structure of 6-MOEG-9-TM-**BF3k** [24] was manipulated by linking the triazole moiety directly to the position 6 of the indene nucleus of 6-MOEG-9-T-**BF3k** (Figure 2) and the resulting benzofulvene monomer was induced to polymerize spontaneously in the apparent absence of initiators and/or catalyst by removal of the solvent to afford PPBFP derivative poly-6-MOEG-9-T-**BF3k**.

Thus as in a “grafting through” approach, the biorelevant PPBFP derivative poly-6-MOEG-9-T-**BF3k** was prepared and characterized in terms of its macromolecular structure, optical features, aggregation liability, and cytocompatibility characteristics. The results of these studies suggest a way to modulate the molecular weight distribution (MWD) and support the evaluation of this PEGylated polybenzofulvene brush in drug delivery applications.

## 2. Materials and Methods 

### 2.1. Synthesis

A Gallenkamp apparatus was used in the determination of melting points in open capillaries. Merck silica gel 60 (230–400 mesh) was used for column chromatography. Merck TLC plates, silica gel 60 F_254_ were used for TLC. NMR spectra were recorded with a Bruker (Karlsruhe, Germany) DRX-400 AVANCE or a Bruker DRX-500 AVANCE spectrometer in the indicated solvents (TMS as internal standard): the values of the chemical shifts are expressed in ppm and the coupling constants (J) in Hz. An Agilent (Santa Clara, CA, USA) 1100 LC/MSD operating with an electrospray source was used in mass spectrometry experiments.

#### 2.1.1. Ethyl 6-[2-(trimethylsilyl)ethynyl]-1-oxo-3-phenyl-1H-indene-2-carboxylate (**2**)

A mixture of triflate derivative **1** [14] (0.40 g, 0.938 mmol) in DMF (10 mL) containing tetrabutylammonium iodide (TBAI, 0.34 g, 0.92 mmol), Pd(PPh_3_)_2_Cl_2_ (0.040 g, 0.057 mmol), CuI (11 mg, 0.058 mmol), TEA (2.0 mL, 14.3 mmol), and ethynyltrimethylsilane (137 mg, 1.39 mmol) was heated at 55 °C under stirring for 1.5 h. The resulting mixture was partitioned between dichloromethane and a saturated solution of NH_4_Cl. The organic layer was dried over sodium sulfate and evaporated under reduced pressure. Purification of the residue by flash chromatography with petroleum ether–ethyl acetate (95:5) as the eluent gave pure compound **2** as an orange solid (0.30 g, yield 85%). An analytical sample was obtained by recrystallization from ethyl acetate by slow evaporation at room temperature (mp 101–103 °C). ^1^H NMR (400 MHz, CDCl_3_): 0.25 (s, 9H), 1.16 (t, *J* = 7.1, 3H), 4.19 (q, *J* = 7.1, 2H), 7.13 (d, *J* = 7.6, 1H), 7.47–7.50 (m, 6H), 7.66 (d, *J* = 1.1, 1H). MS (ESI): *m/z* 397 (M + Na^+^).

#### 2.1.2. Ethyl 6-ethynyl-1-oxo-3-phenyl-1H-indene-2-carboxylate (**3**)

To a mixture of **3** (0.26 g, 0.694 mmol) in absolute ethanol (20 mL) was added a 1M solution of sodium ethoxide in ethanol (1.45 mL, 1.45 mmol) until the complete consumption of **3**. The reaction mixture was then concentrated under reduced pressure and the resulting residue was partitioned between dichloromethane and a saturated solution of sodium chloride. After drying over sodium sulfate, the organic layer was dried and concentrated under reduced pressure. Purification of the residue by flash chromatography with petroleum ether–ethyl acetate (95:5) as the eluent gave **3** as a red solid (0.20 g, yield 95%). Recrystallization from ethyl acetate by slow evaporation of the solvent gave an analytical sample as X-ray quality red-orange crystals melting at 132–134 °C. ^1^H NMR (400 MHz, CDCl_3_): 1.16 (t, *J* = 7.1, 3H), 3.22 (s, 1H), 4.20 (q, *J* = 7.1, 2H), 7.17 (d, *J* = 7.7, 1H), 7.51–7.54 (m, 6H), 7.68 (d, *J* = 1.4, 1H). MS (ESI): *m/z* 325 (M + Na^+^).

#### 2.1.3. Ethyl 6-[1-(2,5,8,11,14,17,20,23,26-nonaoxaoctacosan-28-yl)-1H-1,2,3-triazol-4-yl]-1-oxo-3-phenyl-1H-indene-2-carboxylate (**4**)

A mixture of **3** (0.19 g, 0.629 mmol) in THF (8.0 mL) containing 28-azido-2,5,8,11,14,17,20,23,26-nonaoxaoctacosane [24] (0.14 g, 0.31 mmol), CuBr (0.011 mg, 0.077 mmol), and DIPEA (0.014 mL, 0.080 mmol) was stirred at room temperature for 6 h. The volatile was then removed under reduced pressure and the residue was partitioned between dichloromethane and brine. The organic layer was dried over sodium sulfate and concentrated under reduced pressure. The resulting residue was purified by flash chromatography with ethyl acetate–methanol (9:1) as the eluent. The combined fractions were evaporated under reduced pressure and the residue was dissolved into ethyl acetate and then treated with QUADRASIL MP (100 mg). After filtration the solution was concentrated under reduced pressure to obtain pure compound **4** as a red-orange oil (0.22 g, yield 94%). ^1^H NMR (400 MHz, CDCl_3_): 1.16 (t, *J* = 7.1, 3H), 3.36 (s, 3H), 3.52 (m, 2H), 3.57–3.67 (m, 30H), 3.92 (t, *J* = 5.0, 2H), 4.20 (q, *J* = 7.1, 2H), 4.60 (t, *J* = 4.8, 2H), 7.26 (m, 1H), 7.53 (m, 5H), 7.95 (d, *J* = 1.3, 1H), 8.08 (dd, *J* = 7.6,1.2, 1H), 8.16 (s, 1H). MS (ESI): *m/z* 778 (M + Na^+^).

#### 2.1.4. Ethyl 1-hydroxy-1-methyl-6-[1-(2,5,8,11,14,17,20,23,26-nonaoxaoctacosan-28-yl)-1H-1,2,3-triazol-4-yl]-3-phenyl-1H-indene-2-carboxylate (**5**)

Indenone derivative **4** (0.18 g, 0.238 mmol) was dissolved into dichloromethane (20 mL) and a 2M solution of Al(CH_3_)_3_ in toluene (0.35 mL, 0.70 mmol) was added. The reaction mixture was stirred under an inert atmosphere at room temperature for 20 min, and the Al(CH_3_)_3_ excess was then cautiously destroyed with a 2M NaOH solution. The obtained mixture was partitioned between water and ethyl acetate, and the organic layer was dried over sodium sulfate and concentrated under reduced pressure. Purification of the residue by flash chromatography with dichloromethane–methanol (95:5) as the eluent afforded indenol derivative **5** as a white waxy solid (0.14 g, yield 76%). ^1^H NMR (400 MHz, CDCl_3_): 1.06 (t, *J* = 7.0, 3H), 1.82 (s, 3H), 3.36 (s, 3H), 3.50–3.54 (m, 2H), 3.59–3.65 (m, 30H), 3.69 (s, 1H), 3.92 (t, *J* = 5.0, 2H), 4.07–4.20 (m, 2H), 4.60 (t, *J* = 4.9, 2H), 7.20 (d, *J* = 7.9, 1H), 7.36–7.47 (m, 5H), 7.87 (dd, *J* = 7.9,1.6, 1H), 8.00 (d, *J* = 1.5, 1H), 8.08 (s, 1H). MS (ESI): *m/z* 794 (M + Na^+^).

#### 2.1.5. Ethyl 1-methylene-6-[1-(2,5,8,11,14,17,20,23,26-nonaoxaoctacosan-28-yl)-1H-1,2,3-triazol-4-yl]-3-phenyl-1H-indene-2-carboxylate (6-MOEG-9-T-BF3k)

Procedure A. A mixture of indenol derivative **5** (5.0 mg, 0.00648 mmol) in CDCl_3_ (1.7 mL) and trifluoroacetic acid (TFA, 0.3 mL) containing *p*-toluenesulfonic acid monohydrate (PTSA, 2.0 mg, 0.010 mmol) was refluxed for 30 min and then cooled to room temperature. The reaction mixture was washed with water and dried over sodium sulfate to afford a solution of monomer 6-MOEG-9-T-**BF3k** that was used in the NMR studies. ^1^H NMR (500 MHz, CDCl_3_): 1.06 (t, *J* = 7.0, 3H), 3.36 (s, 3H), 3.52 (m, 2H), 3.57–3.66 (m, 30H), 3.92 (t, *J* = 5.0, 2H), 4.14 (q, *J* = 7.0, 2H), 4.61 (t, *J* = 5.0, 2H), 6.50 (s, 1H), 6.64 (s, 1H), 7.30 (d, *J* = 8.0, 1H), 7.40–7.49 (m, 5H), 7.74 (dd, *J* = 8.0, 1.5, 1H), 8.10 (s, 1H), 8.27 (s, 1H); see Figure 4. ^13^C NMR (500 MHz, CDCl_3_): 13.8, 50.5, 59.0, 60.2, 69.5, 70.5, 71.9, 117.4, 117.8, 121.4, 122.5, 125.7, 128.0, 128.3, 128.7, 130.7, 134.4, 137.6, 141.0, 143.7, 147.6, 152.6, 164.9; see Figure 5.

Procedure B. A mixture of indenol derivative **5** (4.5 mg, 0.00583 mmol) in CD_3_CN (0.75 mL) containing *p*-toluenesulfonic acid monohydrate (0.20 mg, 0.00105 mmol) was heated at 80 °C for 14 h and then cooled to room temperature. The reaction mixture was filtered through a short pad of alumina to afford a solution of monomer 6-MOEG-9-T-**BF3k** that was used in the NMR studies. ^1^H NMR (500 MHz, CD_3_CN): 1.11 (t, *J* = 7.1, 3H), 3.32 (s, 3H), 3.43–3.67 (m, 32H), 3.95 (t, *J* = 5.1, 2H), 4.17 (q, *J* = 7.1, 2H), 4.62 (t, *J* = 5.0, 2H), 6.62 (s, 1H), 6.65 (s, 1H), 7.36 (d, *J* = 7.9, 1H), 7.49–7.58 (m, 5H), 7.90 (dd, *J* = 8.0, 1.5, 1H), 8.34 (s, 1H), 8.38 (d, *J* = 0.9, 1H); see Figure 4. 1^3^C NMR (500 MHz, CDCl_3_): 13.2, 50.2, 57.9, 60.3, 68.9, 69.9, 70.0, 70.1, 71.5, 121.9, 122.4, 125.7, 125.9, 128.2, 128.6, 128.7, 131.2, 134.0, 137.4, 140.5, 143.8, 146.7, 151.7, 164.7; see Figure 5.

#### 2.1.6. Poly[ethyl 1-methylene-6-[1-(2,5,8,11,14,17,20,23,26-nonaoxaoctacosan-28-yl)-1H-1,2,3-triazol-4-yl]-3-phenyl-1H-indene-2-carboxylate] (poly-6-MOEG-9-T-BF3k)

Procedure FE. A mixture of indenol derivative **5** (0.16 g, 0.207 mmol) in CDCl_3_ (18 mL) and trifluoroacetic acid (2.0 mL) containing *p*-toluenesulfonic acid monohydrate (0.055 g, 0.29 mmol) was refluxed for 30 min and then cooled to room temperature. The resulting mixture was then washed with water and dried over sodium sulfate to afford a solution of monomer 6-MOEG-9-T-**BF3k** that was concentrated under reduced pressure. The resulting residue was dissolved into chloroform (10 mL) and again evaporated (this process of dissolution/evaporation was repeated three times). The last residue was purified by precipitation with *n*-hexane from a solution of the polymer in chloroform–ethanol (1:1) and dried under reduced pressure to obtain poly-6-MOEG-9-T-**BF3k**-FE as a gummy pale yellow solid (0.11 g, yield 68% in monomeric unit). ^1^H NMR (500 MHz, CDCl_3_): see Figure 7. ^13^C NMR (125 MHz, CDCl_3_): see Figure 8.

Procedure SE. A mixture of indenol derivative **5** (0.10 g, 0.13 mmol) in CDCl_3_ (10 mL) and trifluoroacetic acid (2.0 mL) containing *p*-toluenesulfonic acid monohydrate (0.036 g, 0.19 mmol) was heated to reflux for 30 min and then cooled to room temperature. The resulting mixture was washed with water and dried over sodium sulfate to afford a solution of monomer 6-MOEG-9-T-**BF3k** that was allowed to concentrate at room temperature by spontaneous evaporation of the solvent. The resulting residue was purified by precipitation with *n*-hexane from a solution of the polymer in dichloromethane and dried under reduced pressure to obtain poly-6-MOEG-9-T-**BF3k**-SE as a gummy pale yellow solid (0.080 g, yield 82% in monomeric unit). ^1^H NMR (500 MHz, CDCl_3_): see Figure 7. ^13^C NMR (125 MHz, CDCl_3_): see Figure 8.

Procedure WA. A mixture of indenol derivative **5** (55 mg, 0.071 mmol) in dry CH_3_CN (10 mL) containing *p*-toluenesulfonic acid monohydrate (2.5 mg, 0.013 mmol) was heated at reflux for 30 h and then cooled to room temperature. The reaction mixture was then filtered through a short pad of alumina to afford a solution of monomer 6-MOEG-9-T-**BF3k** that was diluted with water and concentrated under a gentle nitrogen stream. The resulting colorless mixture was extracted with dichloromethane and the combined organic extracts were dried over sodium sulfate and concentrated under reduced pressure. The resulting residue was purified by precipitation with *n*-hexane from a solution of the polymer in dichloromethane and dried under reduced pressure to obtain poly-6-MOEG-9-T-**BF3k**-WA as a gummy pale yellow solid (16 mg, yield 30% in monomeric unit). ^1^H NMR (500 MHz, CDCl_3_): see Figure 7. ^13^C NMR (125 MHz, CDCl_3_): see Figure 8.

### 2.2. X-ray Crystallography

Single crystals of indenone derivative **3** were used in X-ray data collection on an Oxford-Diffraction Xcalibur Sapphire 3 diffractometer (Santa Clara, CA, USA) equipped with a graphite monochromated Mo Kα radiation (λ = 0.71073 Å) at 293 K. The direct methods implemented in *SHELXS*-2013 program were used in the solution of the structures [37]. The refinements were carried out by full-matrix anisotropic least-squares on F2 for all reflections for non-H atoms by means of the *SHELXL*-2018 program [38]. Crystallographic data (excluding structure factors) for the structure in this paper have been deposited with the Cambridge Crystallographic Data Centre as supplementary publications no. CCDC 1901307 (Supplementary Materials). Copies of the data can be obtained, free of charge, on application to CCDC, 12 Union Road, Cambridge CB2 1EZ, UK; (fax: +44 (0) 1223 336 033; or e-mail: deposit@ccdc.cam.ac.uk).

### 2.3. Mass Spectrometry

MALDI-TOF (matrix assisted laser desorption/ionization-time of flight) mass spectra were recorded in linear mode by means of a Bruker Ultraflex III MALDI-TOF/TOF instrument equipped with a Nd:YAG laser at a wavelength of 355 nm with <500 ps pulse and 200 Hz firing rate. The accelerating voltage was 22 kV. External calibration was performed using a Bruker calibration mixture consisting of polypeptides with different molar mass values. All measurements were performed in positive ion mode. Approximately 2000 laser shots were accumulated for each mass spectrum, and 2,5-dihydroxybenzoic acid was used as the matrix in the analysis of the samples.

Mass spectra in reflectron mode were recorded by means of a 4800 Proteomic Analyzer (Applied Biosystems, Foster City, CA, USA) MALDI-TOF/TOF instrument equipped with a Nd:YAG laser at a wavelength of 355 nm with <500 ps pulse and 200 Hz firing rate. The accelerating voltage was 20 kV. External calibration was performed using an Applied Biosystems calibration mixture consisting of polypeptides with different molar mass values. The irradiance was maintained slightly above the threshold, to obtain a mass resolution of about 7000–10,000 fwhm; isotopic resolution was observed throughout the entire mass range detected (from *m/z* 500 up to *m/z* 5000). Mass accuracy was about 40 ppm. All measurements were performed in positive ion mode; approximately 1500 laser shots were accumulated for each mass spectrum in reflectron mode, and 250 laser shots in linear one too. For the analysis of all the samples several matrices such as DHB, DCTB and dithranol, were used with and without salts as cationizing agent (i.e., CF_3_COOLi; CF_3_COONa). All matrices were solubilized in THF at a concentration of 0.1 M, poly-6-MOEG-9-T-**BF3k** samples were solubilized in the appropriate solvent (i.e., CHCl_3_ or DMF) with a concentration of 2 mg/mL. Samples for MALDI analysis were prepared by the dried-droplet method, in which a mixture of matrix and sample was deposited onto the target plate and dried at room temperature under inert atmosphere (N_2_ flow), and also by the multilayer method, in which firstly a layer of matrix solution (0.3 µL) onto the target plate then after 10 min a layer of polymer solution (0.2 µL) was deposited and finally after 1 h a final layer of matrix solution (0.3 µL) was added. The best spectra were recorded using DCTB as matrix with or without CF_3_COOLi as doping agent. 

### 2.4. SEC-MALS

The MWD (molecular weight distribution) characterization of the polymers was performed by a Wyatt MALS (Santa Barbara, CA, USA) light scattering photometer on-line to a SEC chromatographic system. The SEC-MALS system and the corresponding experimental conditions were identical to those used in our previous studies [9,22,25] and are not reported in detail here.

### 2.5. Dynamic Light Scattering (DLS) Analysis and ζ Potential Measurements

The mean diameter, width of distribution (polydispersity index, PDI), and ζ potential of the nanoparticles were measured at 25 °C using a Zetasizer NanoZS instrument (Worcestershire, UK) fitted with a 532 nm laser at fixed scattering angle of 173°. The intensity-average hydrodynamic diameter (size in nm) and PDI of nanosystems were characterized in double distilled water after 2 and 20 days. Polymer dispersions were obtained at room temperature by stirring samples (0.2–2 mg/mL) until complete dispersion (2 days). The ζ potential (mV) was calculated from the electrophoretic mobility using the Smoluchowsky relationship and assuming that K·a >> 1 (where K and a are the Debye-Hückel parameter and particle radius, respectively). Each experiment was performed in triplicate.

### 2.6. Transmission Electron Microscopy (TEM)

A solution of poly-6-MOEG-9-T-**BF3k** in water was dropped onto a 300 mesh formvar-coated copper grid. The sample excess was then blotted by filter paper and the grids were stained with 1% of aqueous uranyl acetate solution. Samples were observed in a FEI Tecnai G2 Spirit transmission electron microscopy (Hillsboro, OR, USA) at an acceleration voltage of 100 kV.

### 2.7. Cytotoxicity Evaluation

The cell viability was assessed by the tetrazolium salt (MTS) assay on human colon cancer (HCT116) and normal human bronchial epithelial (16HBE) cell lines, (purchased from Istituto Zooprofilattico Sperimentale della Lombardia e dell’Emilia Romagna, Brescia, Italy) using a commercially available kit (Cell Titer 96 Aqueous One Solution Cell Proliferation assay, Promega, Madison, WI, USA ). Cells were seeded in 96-well plates at a density of 10 × 104 cells/well and grown in Dulbecco’s Minimum Essential Medium (DMEM) with 10% FBS (fetal bovine serum) and 1% of penicillin/streptomycin (10,000 U/mL penicillin and 10 mg/mL streptomycin) at 37 °C in 5% CO_2_ humidified atmosphere. After 24 h of cell growth the medium was replaced with 200 μL of fresh culture medium containing poly-6-MOEG-9-T-**BF3k**-FE at the following concentrations per well: 0.005, 0.01, 0.03, 0.1 and 0.5 mg/mL. Cells incubated with fresh culture medium were used as negative control. After 24 and 48 h of incubation time DMEM was replaced with 100 μL of fresh medium and 20 μL of a MTS solution was added to each well. Plates were incubated for an additional 2 h at 37 °C. Then, the absorbance at 490 nm was measured using a microplate reader (Multiskan, Thermo Fisher Scientific Oy, Vantaa, Finland). Pure cell medium was used as a negative control. Results were expressed as percentage reduction of the control cells. All culture experiments were performed in triplicates.

For statistical analysis, a one way analysis of variance (ANOVA) was applied to compare different groups. Data were considered statistically significant with a value of *p* < 0.05 and differences between different groups were compared using a posteriori Bonferroni t-test. All values are expressed as the average of three experiments ± standard deviation.

## 3. Results and Discussion

### 3.1. Synthesis 

**Scheme 1:**
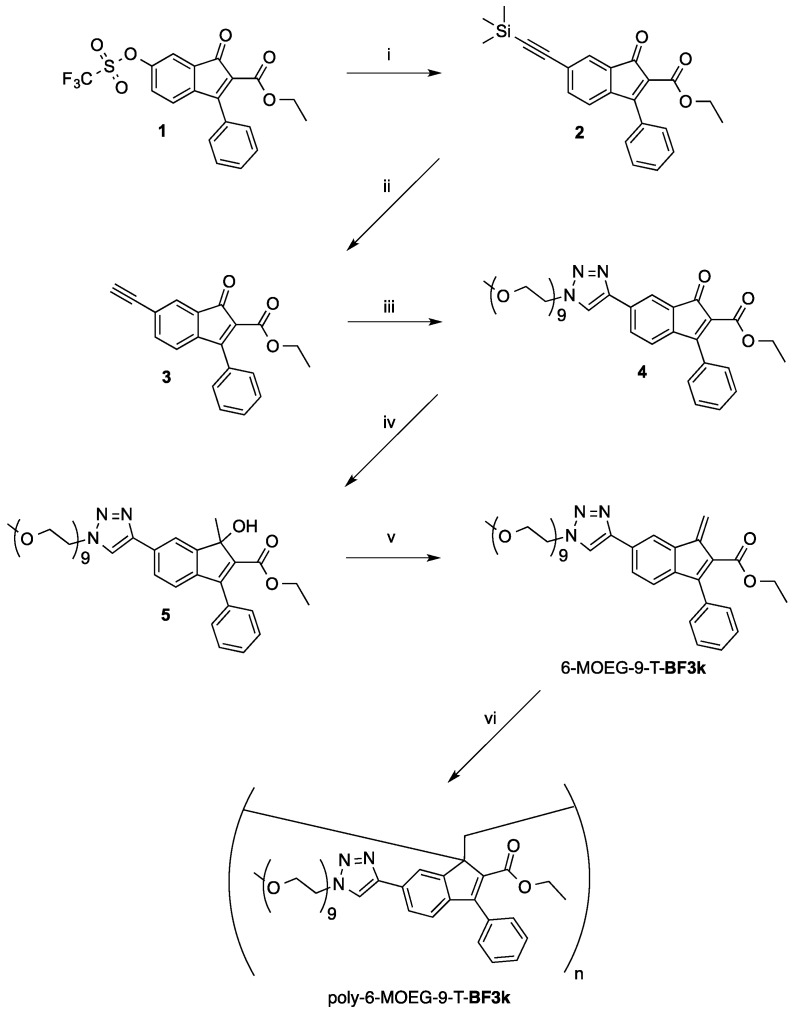
“Grafting through” approach to PPBFB derivative poly-6-MOEG-9-T-BF3k. **Reagents**: (i) ethynyltrimethylsilane, CuI, Pd(PPh_3_)_2_Cl_2_, TBAI, TEA, DMF; (ii) C_2_H_5_ONa, C_2_H_5_OH; (iii) CH_3_(OCH_2_CH_2_)_9_N_3_, CuBr, DIPEA, THF; (iv) Al(CH_3_)_3_, CH_2_Cl_2_; (v) Procedure A: PTSA, TFA, CDCl_3_; Procedure B: PTSA, CD_3_CN; (vi) solvent elimination (for the details see the Materials and Methods section).

The “grafting through” approach to PPBFB derivative poly-6-MOEG-9-T-**BF3k** is shown in Scheme 1. 

**Figure 3:**
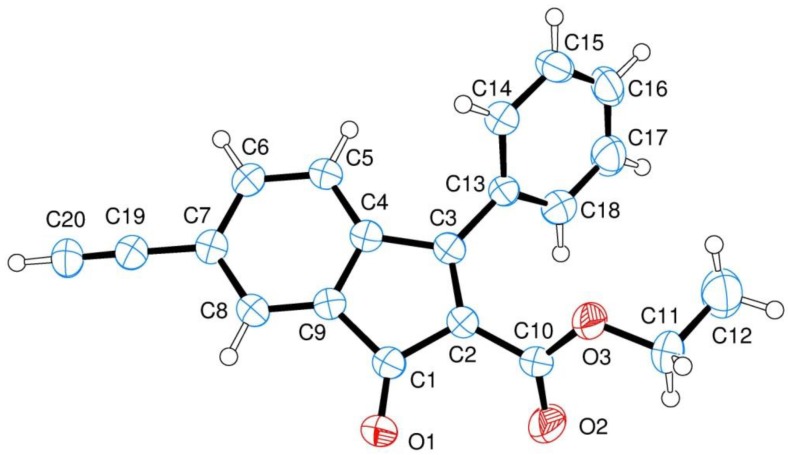
Structure of indenone derivative **3** found by crystallography. Ellipsoids enclose 50% probability.

Sonogashira coupling of triflate **1** [14] with trimethylsilylacetylene (Adrich) gave silyl derivative **2**, which was promptly transformed into the corresponding acetylene derivative **3** by deprotection with sodium ethoxide in ethanol. After confirming the structure of **3** by crystallographic studies (Figure 3), this compound was employed in the CuAAC coupling with MOEG-9 chains bearing an azide group [24] in the presence of CuBr and DIPEA to obtain indenone derivative **4**.

As expected, alkylation of indenone derivative **4** with trimethylaluminium gave indenol **5**, which was dehydrated in chloroform (CDCl_3_, CH_3_CN, or CD_3_CN) in the presence of *p*-toluenesulfonic acid (PTSA) in order to obtain monomer 6-MOEG-9-T-**BF3k**. This benzofulvene derivative was induced to polymerize spontaneously by three different procedures of solvent removal to obtain three different samples of polybenzofulvene derivative poly-6-MOEG-9-T-**BF3k** that were carefully studied by SEC-MALS, NMR spectroscopy, and MALDI-TOF MS techniques (see below). In particular, one polymer sample (i.e., poly-6-MOEG-9-T-**BF3k**-FE) was obtained by the standard procedure consisting of the fast solvent (i.e., CHCl_3_) evaporation at reduced pressure into a rotary evaporator apparatus. The second sample (i.e., poly-6-MOEG-9-T-**BF3k**-SE) was obtained by slow evaporation of the solvent (i.e., CHCl_3_) at room temperature, whereas the third sample (i.e., poly-6-MOEG-9-T-**BF3k**-WA) was obtained by the addition of water to the monomer solution in acetonitrile, followed by the partial evaporation of the organic solvent promoted by a nitrogen stream.

The effective spontaneous polymerization of this benzofulvene monomer in all the tested experimental conditions in the apparent absence of initiators or catalysts supported the efficiency of the recognition step, which we assumed to play a pivotal role in the “affinity polymerization” mechanism [12,13,24]. In particular, the spontaneous polymerization in the water environment suggested that the recognition step is operative also in the water dispersion. Interestingly, we assumed that the presence of MOEG-9 SC in benzofulvene monomer 6-MOEG-9-T-**BF3k** led to the formation of amphiphilic molecules, which could be capable in principle of self-assembling in water solutions to form micelles. The hydrophobic nature of the 3-phenylbenzofulvene moieties could drive the aggregation [39,40,41] leading to the exposition of the MOEG-9 SC to the water environment [42]. In this organization, the recognition process and the following spontaneous polymerization could occur inside the core of the micelles [12,13].

### 3.2. NMR Spectroscopy Studies

**Figure 4:**
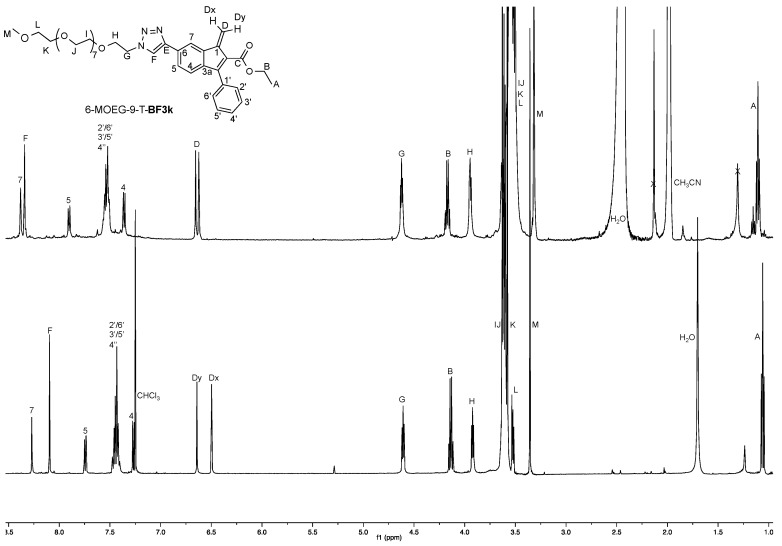
Comparison of the ^1^H NMR spectra (500 MHz) of monomer 6-MOEG-9-T-**BF3k** acquired respectively in CDCl_3_ (bottom trace) and in CD_3_CN (top trace).

**Figure 5:**
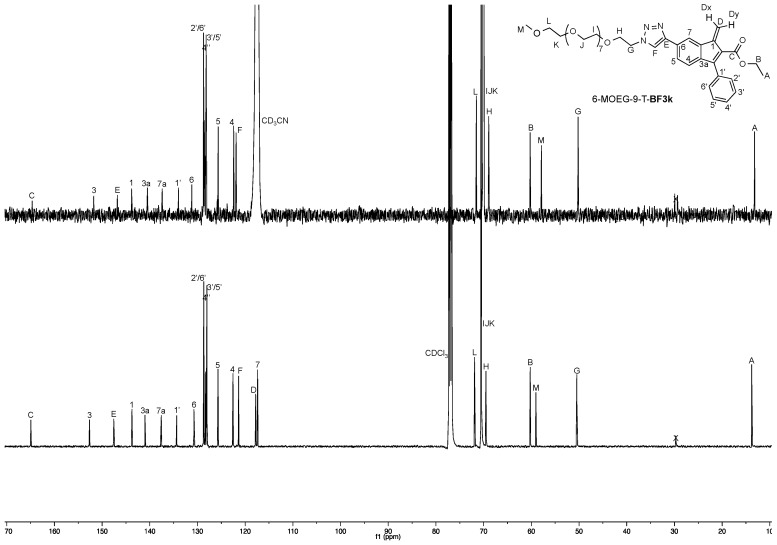
Comparison of the ^13^C NMR spectra (125 MHz) of monomer 6-MOEG-9-T-**BF3k** acquired respectively in CDCl_3_ (bottom trace) and in CD_3_CN (top trace).

The ^1^H NMR spectroscopic features of benzofulvene monomer 6-MOEG-9-T-**BF3k** were studied in different deuterated organic solvents, namely chloroform and acetonitrile (Figure 4 and Figure 5).

**Figure 6:**
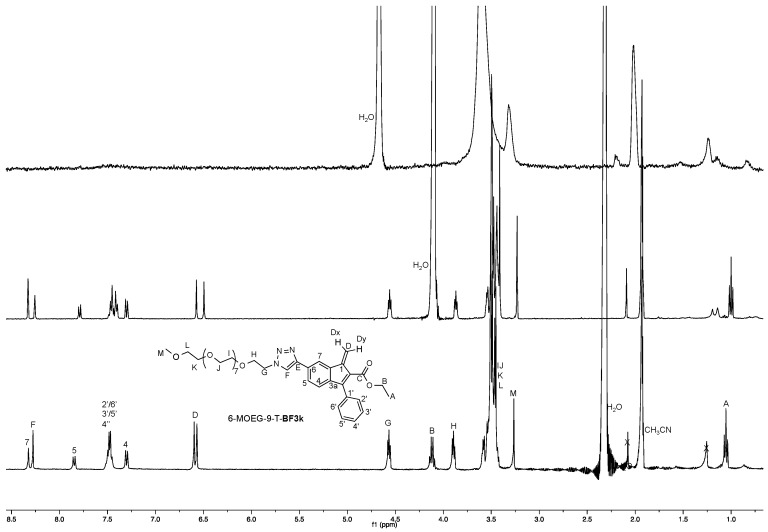
^1^H NMR spectra performed during the transferring of the benzofulvene monomer 6-MOEG-9-T-**BF3k** in the water environment. Comparison of ^1^H NMR spectrum of the benzofulvene monomer in CD_3_CN (bottom trace) with that of the same monomer after diluting (1:1) with D_2_O (middle trace) the initial monomer solution and after the partial evaporation of the organic solvent (top trace).

As expected, the NMR spectra of 6-MOEG-9-T-**BF3k** were dominated by the signals attributed to the MOEG-9 side chain (at around 3.5–3.6 ppm in the ^1^H NMR spectra and at around 70 ppm in the ^13^C NMR spectra). The comparison of the two spectra demonstrated the relatively limited dependence on the solvent system of both the chemical shift values of the signals and the dehydration process, confirming the full equivalence of CHCl_3_ and CH_3_CN in the preparation of this benzofulvene monomer. Thus, the chloroform solutions of the monomer were used for the spontaneous polymerization by solvent evaporation, whereas the acetonitrile solutions were used for transferring the benzofulvene monomer in the water environment. Accordingly, D_2_O was added to the NMR tube containing 6-MOEG-9-T-**BF3k** solution in CH_3_CN doubling the initial volume and the resulting mixture was concentrated by means of a gentle nitrogen stream to the initial volume. This procedure was repeated and we observed that the starting pale yellow solution gradually became a cloudy mixture, suggesting that the pale yellow benzofulvene monomer underwent spontaneous polymerization in the water environment. The ^1^H NMR experiments (Figure 6) performed during the transferring of the benzofulvene monomer in the water environment confirmed the occurrence of the spontaneous polymerization in the water dispersion.

**Figure 7:**
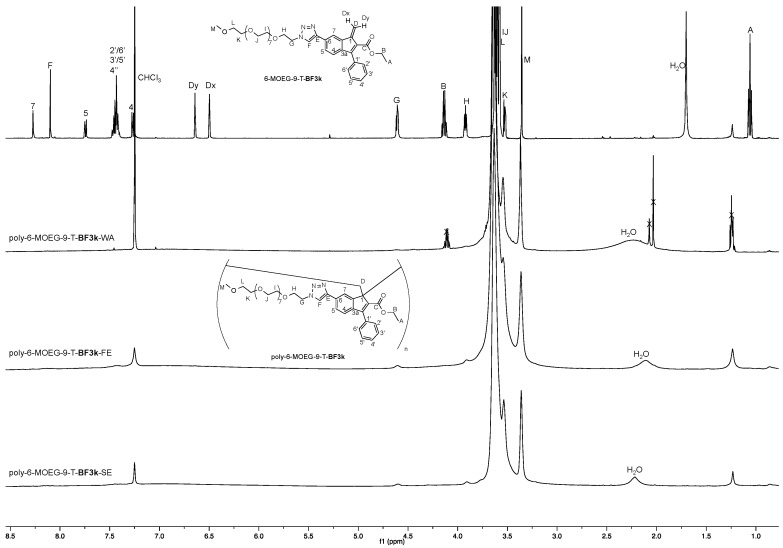
Comparison of the ^1^H NMR spectrum of monomer 6-MOEG-9-T-**BF3k** (500 MHz, CDCl_3_, top trace) with those of the corresponding polymers: poly-6-MOEG-9-T-**BF3k**-WA, poly-6-MOEG-9-T-**BF3k**-FE, and poly-6-MOEG-9-T-**BF3k**-SE.

**Figure 8:**
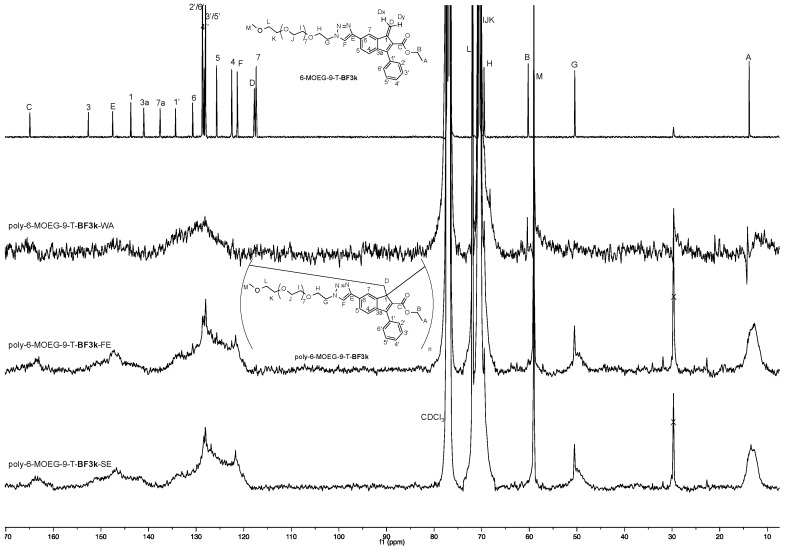
Comparison of the ^13^C NMR spectrum of monomer 6-MOEG-9-T-**BF3k** (125 MHz, CDCl_3_, top trace) with those of the corresponding polymers: poly-6-MOEG-9-T-**BF3k**-WA, poly-6-MOEG-9-T-**BF3k**-FE, and poly-6-MOEG-9-T-**BF3k**-SE.

The polymeric material (i.e., poly-6-MOEG-9-T-**BF3k**-WA) was extracted from the water environment with chloroform and, after purification by precipitation (see Materials and Methods for the details) its NMR spectra were compared with those of poly-6-MOEG-9-T-**BF3k**-FE (i.e., the sample obtained by the standard procedure consisting of the fast solvent evaporation at reduced pressure into a rotary evaporator apparatus) and of poly-6-MOEG-9-T-**BF3k**-SE (i.e., the sample obtained by slow evaporation of the solvent at room temperature (Figure 7 and Figure 8)).

As in the case of monomer 6-MOEG-9-T-**BF3k**, the ^1^H NMR spectra of poly-6-MOEG-9-T-**BF3k** were dominated by the signals at around 3.5–3.6 ppm attributed to the MOEG-9. However, the comparison of the spectra shown in Figure 7 clearly showed that the sharp signals of the monomer became broad in the spectra of the polymers with some up-field shift as already observed in similar polybenzofulvene derivatives. As expected, the aromatic region showed the presence of a single broad signal with a small, but significant shoulder at around 8 ppm attributed to the triazole hydrogen atom.

The ^13^C NMR spectra (Figure 8) showed a higher resolution than that of the ^1^H NMR one, but as observed in similar PEGylated polybenzofulvene brushes [22,23,24,25,26], the MOEG-9 side chains showed a rather negative impact in the NMR features of these polymers (i.e., severe line broadening) and only the direct comparison between monomer and polymer allowed quite reliable analysis to be performed in terms of correspondence between the various peaks. In particular, the presence of broad peaks at around 49 and 57 ppm supported the retention of the spontaneous 1,2-polymerization mechanism as already observed in related PEGylated polybenzofulvene brushes [22,23,24,25,26].

### 3.3. MALDI-TOF Mass Spectrometry Characterization

**Figure 9:**
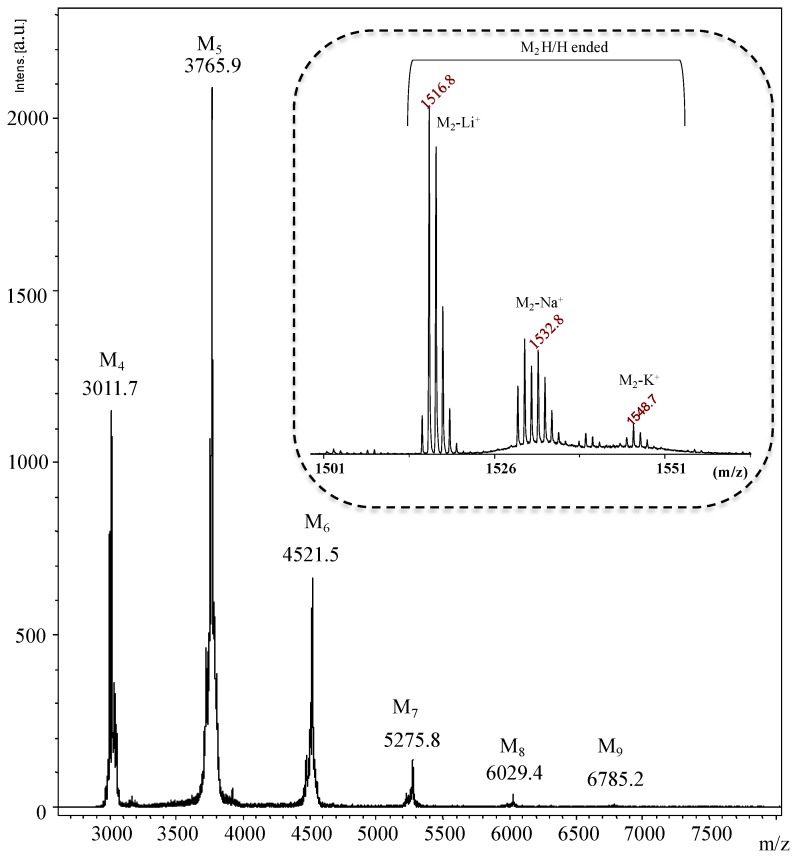
Matrix assisted laser desorption/ionization-time of flight (MALDI-TOF) mass spectrum of poly-6-MOEG-9-T-**BF3k**-FE (i.e., the sample obtained by the standard procedure consisting of the fast solvent evaporation at reduced pressure into a rotary evaporator apparatus). The inset displays an enlarged section of the mass spectrum recorded in reflectron mode using DCTB as matrix and CF_3_COOLi salt as doping agent.

The macromolecular structure of poly-6-MOEG-9-T-**BF3k** was investigated also by MALDI-TOF mass spectrometry. In particular, the MALDI-TOF mass spectrum of poly-6-MOEG-9-T-**BF3k**-FE (i.e., the sample obtained by the standard procedure consisting of the fast solvent evaporation at reduced pressure into a rotary evaporator apparatus, Figure 9) was obtained in linear mode by using 2,5-dihydroxybenzoic acid (DHBA) as the matrix. The best mass spectra in reflectron mode were recorded using DCTB (*trans*-2-[3-(4-*tert*-butylphenyl)-2-methyl-2-propenylidene]malononitrile) as a matrix, often in the presence of Li^+^, Na^+^ and K^+^ salts as doping agent.

As expected, the spectrum showed the presence of families of peaks from *m/z* ~3000 up to *m/z* ~7000 showing 754 Da transitions, which corresponded to the molar mass of the monomeric unit of poly-6-MOEG-9-T-**BF3k** (i.e., 753.8). The main peaks were assigned to the protonated ions of the expected oligomers terminated with hydrogens at both ends (Figure 9). The structure of the polymer chains was also confirmed by MALDI-TOF MS analysis in reflectron which gave more resolved mass peaks and in particular the isotopic distributions of the species composing the samples. The inset in Figure 9 shows the lithiated, sodiated and potassiated ions corresponding to the dimers ended with hydrogen at both ends. The mass spectrum of the poly-6-MOEG-9-T-**BF3k**-WA recorded in reflectron mode was very similar to that of poly-6-MOEG-9-T-**BF3k**-FE and presents in the mass range between 750 and 5000 Da the mass resolved peaks corresponding to the dimers, trimers, tetramers, pentamers and hexamers ended with hydrogen at both ends. On the contrary, very complex mass spectra were recorded by analyzing the poly-6-MOEG-9-T-**BF3k**-SE fraction soluble in CHCl_3_. Besides the peaks due to protonated ions of oligomers terminated with hydrogen, the mass spectra present other mass series corresponding to the oxidized species and to the species maybe formed by reaction of the biradicals with the solvent and or with the MOEG side chains. Therefore, the presence of these new species suggested that during the slow evaporation the biradicals can give cross-linking reactions forming polymer chains insoluble in the common organic solvents used to solubilize the the poly-6-MOEG-9-T-**BF3k** samples (i.e., CHCl_3_, THF, DMSO, DMF). This evidence supports that the better conditions to synthesize the poly-6-MOEG-9-T-**BF3k** samples occurs at very fast evaporation of the solvent (i.e., CHCl_3_).

### 3.4. Molecular Weight Distribution Characterization

The molecular weight distribution in poly-6-MOEG-9-T-**BF3k** samples (i.e., poly-6-MOEG-9-T-**BF3k**-WA, poly-6-MOEG-9-T-**BF3k**-FE, and poly-6-MOEG-9-T-**BF3k**-SE) was analyzed by means of a SEC-MALS system with THF–DMF (8:2) containing 0.01 M LiBr as the mobile phase. 

Very interestingly, the results summarized in Table 1 proved that the MWD features of poly-6-MOEG-9-T-**BF3k** samples were affected by the polymerization conditions. In fact, the MWD of the sample obtained by the standard procedure consisting of the fast solvent evaporation at reduced pressure into a rotary evaporator apparatus (i.e., poly-6-MOEG-9-T-**BF3k**-FE) showed *M*_p_ and polydispersity index *M*_W_/*M*_n_ values in the same range observed for previously published PPBFB poly-6-MOEG-9-TM-**BF3k** and poly-6-MOEG-9-**BF3k**, which were included in Table 1 for comparative purposes [23,24,25]. 

On the other hand, the sample obtained by slow evaporation of the solvent at room temperature (i.e., poly-6-MOEG-9-T-**BF3k**-SE) showed a surprisingly low *M*_p_ value, which was around two orders of magnitude lower than the one shown by poly-6-MOEG-9-T-**BF3k**-SE. Accordingly, the polydispersity index *M*_W_/*M*_n_ value was also the lowest measured for these PPBFB derivatives. Moreover, the sample obtained by spontaneous polymerization in a water environment (i.e., poly-6-MOEG-9-T-**BF3k**-WA) showed intermediate MWD features. 

We assume that the slow evaporation of the solvent at room temperature (as in a typical crystallization procedure) induces 6-MOEG-9-T-**BF3k** monomers to aggregate slowly in columnar aggregates, which undergo spontaneous polymerization when a critical number of monomers are compacted to reach the critical stereo-electronic parameters for the establishment of the covalent bonds leading to the polymer backbone. The spontaneous polymerization was rapidly terminated by the interaction of the polymeric biradical species with the excess of the quenching ones (i.e., water molecules) present in the polymerization mixture. Thus, the macromolecules contained in poly-6-MOEG-9-T-**BF3k**-SE sample could be assumed to represent the true initiator species in the spontaneous polymerization of this benzofulvene monomer. These initiator species could develop into a polymer showing an intermediate molecular weight distribution, in the presence of a limited number of monomer molecules composing the micelles that are assumed to be involved in the formation of poly-6-MOEG-9-T-**BF3k**-WA sample. On the other hand, when a large number of monomer molecules are condensed in a small space and the quenching species are rare as during the fast solvent evaporation at reduced pressure into a rotary evaporator apparatus, the small initiator species could easily evolve into polybenzofulvene macromolecules (i.e., poly-6-MOEG-9-T-**BF3k**-FE) showing a relatively high molecular weight and corresponding to an apparent plateau very similar in the related PPBFB derivatives reported in Table 1. 

Apart from the mechanistic considerations, these observations indicate a possible way to modulate the MWD of these PPBFB derivatives by spontaneous polymerization.

### 3.5. Self-Aggregating Properties 

**Figure 10:**
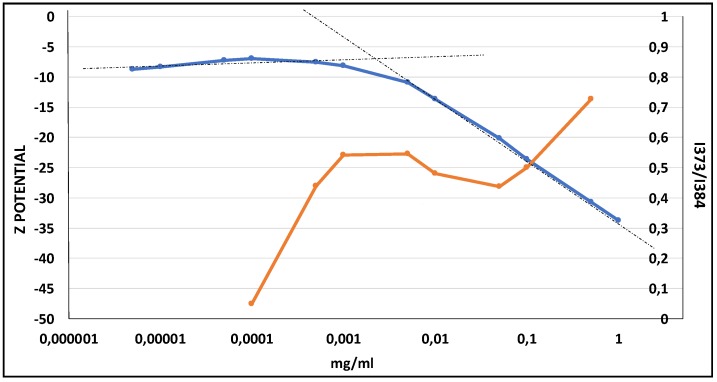
I_373_/I_384_ intensity ratio obtained from pyrene emission spectra in the presence of poly-6-MOEG-9-T-**BF3k**-FE (blue curve) and Z-potential values (orange curve) as a function of polymer concentration.

The ability of PPBFB derivative poly-6-MOEG-9-T-**BF3k** to generate supramolecular carriers spontaneously was evaluated by critical aggregation concentration (CAC) determinations (i.e., pyrene fluorescence analysis) and then by means of dynamic light scattering analysis and zeta potential measurements. In particular, the CAC value was calculated from the intersection of the regression lines obtained before and after the slope change (low and high concentration regions, Figure 10). Among the newly-synthesized PPBFB samples, only poly-6-MOEG-9-T-**BF3k**-FE showed a CAC value between 0.001 and 0.05 mg/mL. Interestingly, the variations of I_373_/I_384_ intensity ratio obtained from pyrene emission spectra were in accordance with the stabilization of the surface Z potential values, correlated to the generation of a homogeneous self-aggregating nanoparticles population.

Thus, DLS analysis was performed on poly-6-MOEG-9-T-**BF3k**-FE samples, which were dispersed in ultrapure water at room temperature at concentration values higher than the found CMC one. DLS measurements were performed after 2 and 30 days and no mean variation in size was observed during this time frame (Table 2).

**Figure 11:**
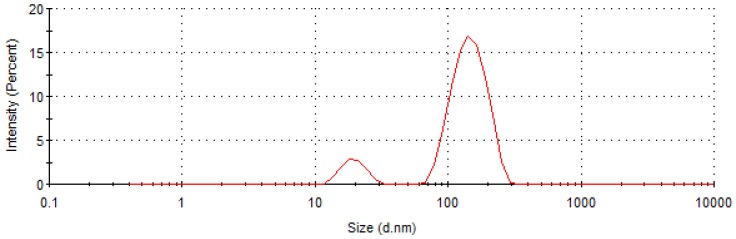
DLS size distribution histograms of poly-6-MOEG-9-T-**BF3k**-FE dispersions in ultrapure water.

The results advocated that poly-6-MOEG-9-T-**BF3k**-FE was inclined to produce water-soluble nanoparticles showing dimensions around 166 nm in agreement with the results obtained with parent PPBFB derivative poly-6-MOEG-9-TM-**BF3k** samples [24,25]. In particular, poly-6-MOEG-9-T-**BF3k**-FE derivative generated in water two homogeneous populations (Figure 11), which, on the bases of the apparent dimensions, were assumed to be isolated macromolecules (diameter around 20 nm) and supra-macromolecular aggregates (diameter around 150 nm). 

### 3.6. Transmission Electron Microscopy Studies

**Figure 12:**
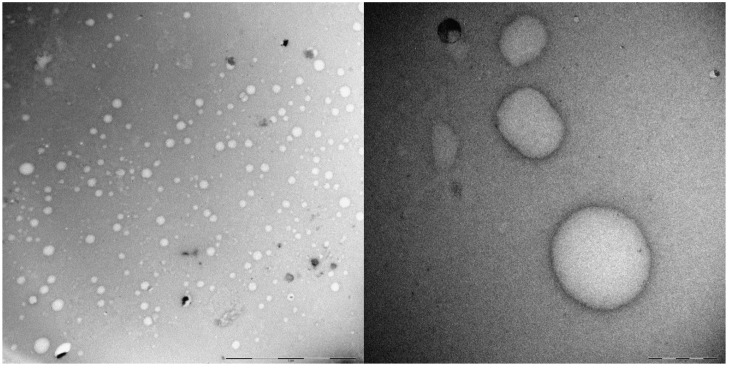
Structure of macromolecular aggregates found by TEM analysis of poly-6-MOEG-9-T-**BF3k**-FE solutions in water. The scale bar corresponds to 1 μm in the left panel and 200 nm in the right panel.

The self-aggregating features of poly-6-MOEG-9-T-**BF3k**-FE were substantiated by the results of TEM analysis. In particular, a sample (~2 mg) of the polymer was treated with water (0.5 mL) at room temperature in static conditions to produce the swelling of the polymer in few minutes to form a transparent hydrogel, which was expanded by the progressive swelling up the formation of an opalescent dispersion after 1 h. After equilibration at room temperature for two weeks, the nearly transparent dispersion was analyzed by transmission electron microscopy (TEM) with negative staining with uranyl acetate. The analysis of TEM images suggested the propensity of poly-6-MOEG-9-T-**BF3k**-FE to generate spherical objects showing dimensions in the range of 20–200 nm (Figure 12) in agreement with the assumption that isolated macromolecules (~20 nm) were liable to self-assemble into supramolecular entities showing greater dimensions.

### 3.7. Photophysical Characterization

**Figure 13:**
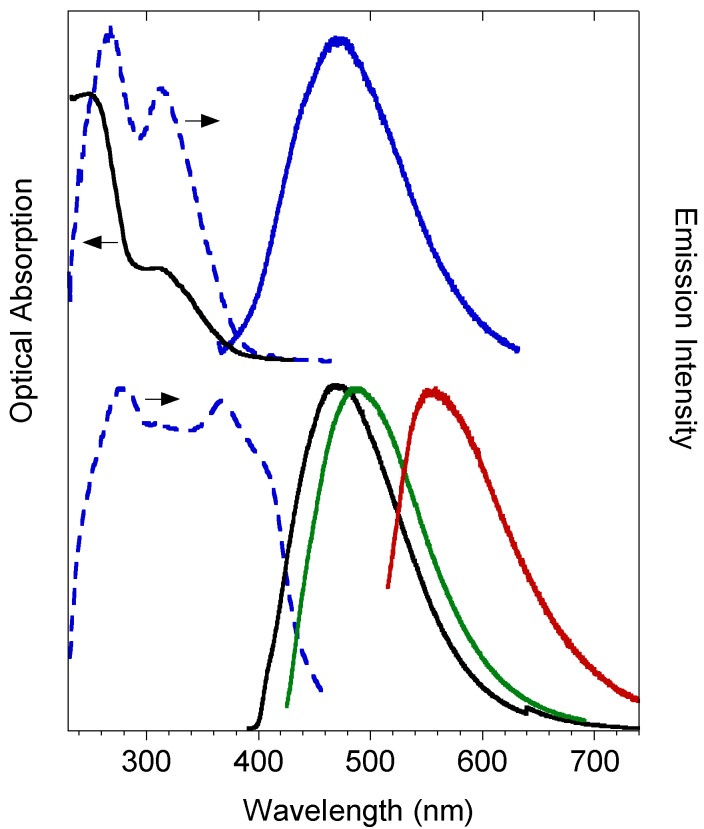
Normalized absorption and emission spectra of poly-6-MOEG-9-T-**BF3k**-FE in dichloromethane solution (top) and in the solid state (bottom). Optical absorption (black solid line), emission excitation profiles (λ_em_ = 470 nm, dotted blue lines) and emission spectra (λ_ex_ = 330 nm, blue solid line; λ_ex_ = 390 nm, black solid line; λ_ex_ = 420 nm, green solid line; λ_ex_ = 490 nm, red solid line).

The development of efficient drug delivery systems requires the understanding of their interactions with the biological systems (e.g., cell/tissue penetration, interaction with possible targets, metabolism). In general, the emission properties of both small molecules or polymeric materials are largely exploited in biological investigations as well as in some clinical applications. In particular, the visible portion of the radiative spectrum (400–800 nm) is widely used in fluorescence microscopy, which represents a valuable tool for the investigation of many functional aspects of drug delivery systems [43]. The polybenzofulvene derivatives represent interesting π-stacked fluorescent polymers capable of emitting visible light [14,18,23,24]. We assumed that in poly-6-MOEG-9-T-**BF3k**-FE, the insertion of a triazole ring directly connected to the 3-phenylindene structure can increase conjugation in the repeating unit and therefore improve the optical features useful in fluorescence microscopy studies. Thus, the photophysical features of PPBFB derivative poly-6-MOEG-9-T-**BF3k**-FE were studied both in solution and in the solid state (i.e., gummy solid) (Figure 13).

The diluted dichloromethane solution displays two absorption bands at about 315 and 250 nm with a blue emission (λ_em_ = 470 nm) showing a quantum yield (QY) of 4%. The emission in the solid state is characterized by a very similar blue broad band, peaked at 470 nm, that displays a higher QY of 17% (λ_ex_ = 380 nm). Interestingly, by selective excitation at longer wavelengths, a red-shift of the emission band is detected at 490 nm (λ_ex_ = 420 nm), down to 555 nm (λ_ex_ = 490 nm). This behavior indicates that in the solid state the polymeric chains display different aggregation motives, providing a wide emission in the blue-green visible region. Interesting, the absorption/emission features and the significant QY of poly-6-MOEG-9-T-**BF3k**-FE can be considered compatible with its employment in a fluorescence microscopy apparatus using standard filters for fluorescence stain DAPI (4′-6-diamidino-2-phenylindole) [44,45].

### 3.8. Biocompatibility Evaluation

**Figure 14:**
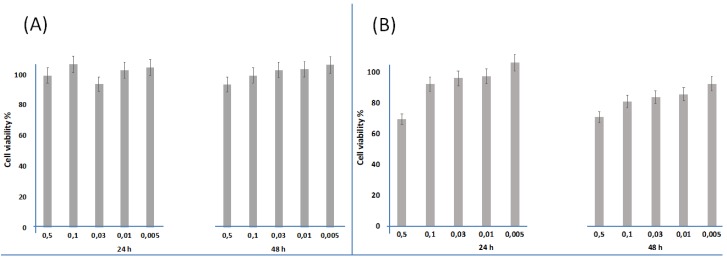
Cell viability % (tetrazolium salt (MTS) assay) of poly-6-MOEG-9-T-**BF3k**-FE on human colon cancer cells HCT116 (panel **A**) and normal human bronchial epithelial cells 16HBE (panel **B**) cells, after 24 h and 48 h of incubation at concentrations of 0.005, 0.01, 0.03, 0.1 and 0.5 mg/mL.

The potential cytotoxicity features of PPBFB derivative poly-6-MOEG-9-T-**BF3k-**FE were evaluated both on human colon adenocarcinoma HCT116 cells and on normal human bronchial epithelial (16HBE) cell lines, the latter being widely used to model barrier function of the airway epithelium, after incubation for 24 and 48 h. The tested concentrations ranged from 0.005 to 0.5 mg/mL, and the results (Figure 14) demonstrated that, for both incubation times, 24 h and 48 h, the cell viability was always comparable to the control cells. Even on normal bronchial epithelial cells, notoriously very sensitive to xenobiotic agents and largely used to test biocompatibility on normal human cells [46,47,48], the tested polymer resulted to be sufficiently biocompatible with the exception of the highest concentration of 0.5 mg/mL. Therefore, poly-6-MOEG-9-T-**BF3k-**FE derivative was proven to be clearly cytocompatible at incubation concentrations below 0.5 mg/mL; actually, below this value cell viability is always above 80%, even for normal cells. This result supports its potential employment in nanoencapsulation and delivery of drug molecules. 

## 4. Conclusions

A novel PEGylated polybenzofulvene brush (i.e., poly-6-MOEG-9-T-**BF3k**) was prepared by means of a “grafting through” approach in which the polybenzofulvene backbone was built by spontaneous polymerization of benzofulvene macromonomer 6-MOEG-9-T-**BF3k** bearing a MOEG-9 side chain linked to position 6 of the 3-phenylindene scaffold by means of a triazole heterocycle. The spontaneous polymerization of the benzofulvene macromonomer was assayed in three different procedures of solvent removal and the successful spontaneous polymerization in all the experimental conditions supported the efficiency of the recognition step at the base of the assumed “affinity polymerization” mechanism. Very intriguingly, the spontaneous polymerization occurred also in a water environment, where benzofulvene monomer 6-MOEG-9-T-**BF3k** was assumed to self-assemble to form micelles. The three polymer samples were characterized by SEC-MALS, NMR spectroscopy, and MALDI-TOF MS techniques, and the differences observed in the MWD features suggested the spontaneous polymerization could advance through the formation of oligomeric initiatory species, which evolve in macromolecules showing different polymerization degrees depending on the presence of monomers molecules and/or quenching species. 

The high molecular weight poly-6-MOEG-9-T-**BF3k** was then characterized from the point of view of its aggregation liability and potential biocompatibility. In particular, pyrene fluorescence analysis suggested a CAC value between 0.01 and 0.05 mg/mL, and DLS studies showed the presence in the water dispersions of two homogeneous populations: one showing dimensions around 20 nm and the other showing diameters around 150 nm. This result was confirmed by TEM analysis, which showed the presence of spherical objects showing dimensions in the range of 20–200 nm. On the basis of their apparent dimensions, these objects were assumed to be isolated macromolecules and supra-macromolecular aggregates. Interestingly, the cytocompatibility shown by poly-6-MOEG-9-T-**BF3k**-FE at concentrations below 0.5 mg/mL, taken together with its aggregation features, supports its potential employment in nanoencapsulation and delivery of drug molecules. Moreover, the emission features of this PPBFB derivative constituted an added value owing to the possibility of monitoring the biodistribution by fluorescence microscopy techniques. 

In particular, the property of the polymer of spontaneously forming polymeric micelles in water makes it an ideal candidate for the encapsulation of drugs that are low soluble in water and with a high therapeutic index, e.g., many anticancer drugs. Furthermore, the colloidal dimensions of the nanosystems it constitutes should, as is known, allow an accumulation into tumor masses thanks to the well-known enhanced permeation and retention effect, without excluding the possibility of monitoring the accumulation of the encapsulated drug by exploiting the fluorescence emission properties of the polymer.

## Figures and Tables

**Table 1:** Macromolecular features of the newly-synthesized poly-6-MOEG-9-T-**BF3k** samples in comparison with the ones shown by previously described poly-6-MOEG-9-TM-**BF3k**, and poly-6-MOEG-9-**BF3k**.

Polymer	*M*_p_ (kg/mol) ^a^	*M*_W_ (kg/mol) ^b^	PDI ^c^	*R*_g_ (nm) ^d^
poly-6-MOEG-9-T-**BF3k**-SE	5.14	6.05	1.5	-
poly-6-MOEG-9-T-**BF3k**-WA	63.8	73.4	2.1	19.0
poly-6-MOEG-9-T-**BF3k**-FE	488	752	2.5	41.3
poly-6-MOEG-9-TM-**BF3k**-GO ^e^	484	821	8.9	33.6
poly-6-MOEG-9-TM-**BF3k**-GT ^e^	193	358	3.9	15.5
poly-6-MOEG-9-**BF3k**	527	456	1.8	20.8

^a^*M*_p_: peak molecular weight; ^b^*M*_w_: weight-average molecular weight; ^c^ PDI: polydispersity index, PDI = *M*_w_/*M*_n_ where *M*_n_ denotes the number-average molecular weight; ^d^ *R*_g_: radius of gyration, which is the dimension of the macromolecules. ^e^ Poly-6-MOEG-9-TM-**BF3k**-GT sample was obtained by the grafting through approach, whereas poly-6-MOEG-9-TM-**BF3k**-GO was prepared by the grafting onto method [24,25].

**Table 2:** Dynamic light scattering (DLS) results obtained with poly-6-MOEG-9-T-**BF3k**-FE in ultrapure water at 25 °C (reported data are those recorded after 2 days) compared with those obtained with parent PPBFB derivative poly-6-MOEG-9-TM-**BF3k** samples.

Polymer Sample	Za (nm ± SD)	PDI ± SD	Zp (mV ± SD)
poly-6-MOEG-9-T-**BF3k**-FE	166 ± 15	0.24 ± 0.02	−26.0 ± 7.2
poly-6-MOEG-9-TM-**BF3k**-GT ^a^	245 ± 25	0.43 ± 0.04	-
poly-6-MOEG-9-TM-**BF3k**-GO ^a^	57 ± 6	0.38 ± 0.04	−7.23 ± 1.5

^a^ Poly-6-MOEG-9-TM-**BF3k**-GT sample was obtained by the grafting through approach, whereas poly-6-MOEG-9-TM-**BF3k**-GO was prepared by the grafting onto method [24,25].

## Supplementary Materials

The following are available online at https://www.mdpi.com/1999-4923/11/9/444/s1, the CIF and the checkCIF output files.
